# Effects of spatial variation in dose delivery: what can we learn from radon-related lung cancer studies?

**DOI:** 10.1007/s00411-022-00998-y

**Published:** 2022-10-08

**Authors:** Balázs G. Madas, Jan Boei, Nora Fenske, Werner Hofmann, Laura Mezquita

**Affiliations:** 1 Environmental Physics Department, Centre for Energy Research, Budapest, Hungary; 2 Department of Human Genetics, Leiden University Medical Center, Leiden, The Netherlands; 3 Federal Office for Radiation Protection, Munich (Neuherberg), Germany; 4 Biological Physics, Department of Chemistry and Physics of Materials, University of Salzburg, Salzburg, Austria; 5 Medical Oncology Department, Hospital Clinic of Barcelona, Barcelona, Spain; 6 Laboratory of Translational Genomic and Targeted Therapies in Solid Tumors, IDIBAPS, Barcelona, Spain

**Keywords:** Carcinogenesis, Dosimetry, Mathematical modelling, Molecular mechanisms, Radon exposure, Risk assessment

## Abstract

Exposure to radon progeny results in heterogeneous dose distributions in many different spatial scales. The aim of this review is to provide an overview on the state of the art in epidemiology, clinical observations, cell biology, dosimetry, and modelling related to radon exposure and its association with lung cancer, along with priorities for future research. Particular attention is paid on the effects of spatial variation in dose delivery within the organs, a factor not considered in radiation protection. It is concluded that a multidisciplinary approach is required to improve risk assessment and mechanistic understanding of carcinogenesis related to radon exposure. To achieve these goals, important steps would be to clarify whether radon can cause other diseases than lung cancer, and to investigate radon-related health risks in children or persons at young ages. Also, a better understanding of the combined effects of radon and smoking is needed, which can be achieved by integrating epidemiological, clinical, pathological, and molecular oncology data to obtain a radon-associated signature. While in vitro models derived from primary human bronchial epithelial cells can help to identify new and corroborate existing biomarkers, they also allow to study the effects of heterogeneous dose distributions including the effects of locally high doses. These novel approaches can provide valuable input and validation data for mathematical models for risk assessment. These models can be applied to quantitatively translate the knowledge obtained from radon exposure to other exposures resulting in heterogeneous dose distributions within an organ to support radiation protection in general.

## Introduction

Understanding the health effects of radon exposure is one of the most important topics in radiation protection. Radon exposure is considered to be one of the most important causes of lung cancer after smoking (WHO 2021). In terms of effective dose, it provides around 50% of the natural background radiation (UNSCEAR 2000). This also means that both exposure and effect are prevalent enough to provide valuable information on the dose–effect relationship for ionizing radiation in general.

Considering the effects of spatial variation in dose delivery, exposure to radon progeny results in heterogeneous dose distributions in many different spatial scales. The dose distribution is highly heterogeneous at the organism level with the lungs being the most exposed organ. The deposition of radon progeny within the lungs is heterogeneous as well resulting in a highly heterogeneous dose distribution within the most affected organ. Finally, alpha particles with high Linear Energy Transfer are responsible for around 97% of total absorbed dose from radon exposure (Markovic et al. 2011) resulting in a subcellular heterogeneity in energy deposition. Besides the spatial variation in dose delivery, the effect of temporal variation is also interesting in case of radon exposure. Epidemiological studies suggest that the excess risk per unit of radon exposure is larger for protracted low-level exposure than for acute high-level exposure (also called inverse exposure-rate effect, UNSCEAR 2020).

These characteristics of radon exposure make it highly relevant regarding the effects of spatial and temporal variation in dose delivery. The aim of this review is to provide an overview on the state of the art in epidemiology, clinical observations, cell biology, dosimetry, and modelling related to radon exposure along with priorities for future research. As the effects of spatial heterogeneity in energy deposition (Baiocco et al. 2022), partial body exposures (Pazzaglia et al. 2022), and dose rate (Lowe et al. 2022) are covered in other papers of this theme issue, particular attention is paid here on the effects of spatial variation in dose delivery in the lungs upon radon exposure, a factor not considered in radiation protection (Madas 2016a).

## Epidemiology

Epidemiological studies on radon mainly comprise uranium miner studies and residential radon studies. Both have clearly shown that inhalation of radon and its decay products can cause lung cancer. Other health effects have not been consistently demonstrated.

### Uranium miner studies

Cohort studies with uranium miners have been conducted since the 1950s to investigate health risks from radon in occupational exposure situations with very high to low levels of radon (NRC 1999a; Laurier et al. 2020). Based on these studies, an increased mortality from lung cancer due to radon exposure was demonstrated. Then, in 1988, radon was classified as a human carcinogen by the International Agency for Research on Cancer (IARC) of the World Health Organization (WHO) (IARC 1988).

An important milestone regarding uranium miner studies was the joint analysis of 11 miner studies in the 1990s (Lubin et al. 1994, 1995; NRC 1999a). This analysis included more than 60,000 miners of 11 cohort studies from Europe, North America, Asia, and Australia, of whom nearly 2700 died from lung cancer. The main findings were a linear increase in lung cancer risk with increasing radon exposure; more specifically, a statistically significant increase in excess relative risk (ERR) for lung cancer mortality per unit of cumulative radon exposure in working level months^1^ (WLM) was shown. Furthermore, based on advanced statistical modelling, a modification of this linear relationship by attained age, time since exposure and exposure rate was found, meaning that the slope of the linear exposure-effect relationship decreases with increasing attained age, increasing time since exposure, and increasing exposure rate. This inverse exposure-rate effect indicates that, given the same total cumulative radon exposure, chronic exposure to low radon concentrations over a longer period of time are associated with a higher lung cancer risk than high radon exposures accumulated over a comparably short period of time (NRC 1999a; Laurier et al. 2020).

Since then, most studies have been updated (see UNSCEAR 2020 for a summary) and a large cohort study with German uranium miners (58,974 miners, 3942 lung cancer deaths) has been added (Kreuzer et al. 2010, 2018). This latter study allowed for an independent evaluation of the joint analysis of 11 miner studies and, overall, confirmed the results (linear relationship of radon-related lung cancer risk with effect modification by time since exposure, attained age/age at exposure and exposure rate). In addition, a statistically significant linear increase in lung cancer risk was shown also for moderate to low levels of radon (below 50–100 WLM). However, the inverse exposure-rate effect is no longer observed at low levels of cumulative radon exposure (Laurier et al. 2020).

Currently, the pooled uranium miners analysis (PUMA) is ongoing (Rage et al. 2020; Richardson et al. 2021). In this project, data from almost 125,000 uranium miners from 7 cohort studies from Europe (Czech Republic, France, Germany) and North America (Canada, USA) are pooled and jointly analysed. Based on the considerably increased data base compared with previous pooled studies, the PUMA study allows for a more detailed investigation of radon-related health risks. Estimates of the radon-related lung cancer risk among relatively contemporary miners in PUMA are coherent with previous results (Richardson et al. 2022).

### Residential radon studies

Since the 1980s, it was investigated whether exposure to low radon concentrations, such as those typically present indoors, also involves a health risk to the population. Findings from miner studies were not directly transferable to the general population as all miners were males, performed heavy physical work and had non-representative smoking patterns. Therefore, numerous case–control studies on residential radon and lung cancer have been conducted since then. Often, these single studies did not have sufficient statistical power and led to ambiguous results.

A series of pooled case–control studies on residential radon, however, consistently showed a linear increase in lung cancer risk with increasing long-term residential radon exposure (Darby et al. 2005, 2006; Krewski et al. 2005, 2006; Lubin et al. 2004). In these studies, individual radon exposure was quantified as a time-weighted average of measured radon concentrations in the current and previously occupied homes of the study participants. In the large European pooled analysis (Darby et al. 2005, 2006), the ERR per 100 Bq/m^3^ was estimated to be 16% and there was no evidence of a threshold value below which radon exposure presents no risk. Even when restricting the analyses to study participants with long-term radon concentrations in their homes below 200 Bq/m^3^ or to lifelong non-smokers, the linear increase in the radon-related lung cancer risk remained statistically significant. Thus, even small radon exposures can slightly increase the risk of lung cancer.

### Radon and smoking

Smoking is the most important risk factor for lung cancer. In residential radon studies, detailed individual information on smoking was collected and all risk estimates were adjusted for smoking. In uranium miner studies, usually only coarse information on smoking is available, however, studies involving this information clearly demonstrated that smoking is no major confounder in this group. In examining the combined effects of smoking and radon on lung cancer risk, both miner studies and residential radon studies showed that radon increases the risk of lung cancer for all persons: current smokers, ex-smokers, and lifelong non-smokers (never-smokers). However, the absolute lung cancer risk due to radon for smokers and ex-smokers is higher than that of never-smokers. Residential radon studies as well as miner studies suggest a (sub-)multiplicative interaction between radon and smoking on the lung cancer risk (e.g. Darby et al. 2005, 2006; Kreuzer et al. 2018; Leuraud et al. 2011; UNSCEAR 2020). That is, radon and smoking mutually increase each other’s effect on lung cancer. Therefore, radon is more likely to cause lung cancer in persons who smoke or have smoked in the past compared to lifelong non-smokers. At the same time, radon exposure is one of the leading causes of lung cancer in persons who have never smoked.

Further variables with potential impact on the association between radon and lung cancer are sex (as potential effect modifier) and other co-exposures (as potential confounders), such as in occupational settings external gamma radiation, uranium ore dust, arsenic or silica dust, diesel exhaust or asbestos. Data on such co-exposures are generally scarce in uranium mining studies. Several studies included such factors (Sogl et al. 2012; Walsh et al. 2015; Rage et al. 2015), but in general, the linear increase in lung cancer risk with increasing radon exposure remained even after adjustment.

### Priorities for new research activities

While there is clear evidence that radon can cause lung cancer, it has not yet been conclusively clarified whether radon can cause other diseases and specifically other cancers than lung cancer. Absorbed doses from inhaled radon progeny to organs other than lung and respiratory tract tend to be substantially lower than absorbed doses to the lung (Marsh et al. 2021). For this reason, it is expected that if there is an excess risk, this risk is considerably lower than the risk for lung cancer. Thus, it can only be observed in large studies and at high radon exposures.

To date, radon epidemiology has focused on modelling exposure–response relationships. Lung cancer risks are related to radon exposure in WLM in uranium miner studies or to radon exposure quantified as long-term average radon concentration in Bq/m^3^ in residential radon studies. It would be of huge interest to model dose–response relationships directly to investigate radon-related health risks, both for the lung and other organs. For this purpose, dosimetric protocols need to be developed to provide data on absorbed organ doses and their distributions in epidemiological studies.

At present, radon exposures and doses as well as radon-related health risk estimates in epidemiological studies involve numerous uncertainties, and further investigation is required to reduce them. Refined modelling of temporal effect modifiers (age at and time since exposure) and exposure-rate effects would be of huge interest. Also combined effects of radon and sex, as well as radon and other co-exposures, particularly smoking, need further clarification. In addition, radon-related health risks should be further investigated at low radon exposures and exposure rates (UNSCEAR 2020). Further research is also needed to investigate radon-related health risks in children or persons exposed at young ages. To date, only very little is known about this and the data available are insufficient (UNSCEAR, 2013).

There are various international research projects ongoing to investigate these questions, such as PUMA or the large European RadoNorm project (Managing risks from radon and NORM, towards effective radiation protection based on improved scientific evidence and social considerations—focus on Radon and NORM, http://www.radonorm.eu).

## Clinical observations

In most epidemiological studies, lung cancer is considered as a unique disease (Pavia et al. 2003; Darby et al. 2005). Due to substantial improvements in our understanding of lung cancer in the past two decades, however, it is not considered as a unique entity anymore but currently divided into a wide range of different subtypes with different clinical, pathological, and molecular characteristics (Thai et al. 2021). All the advances made in diagnosis and treatments have led to improved prognosis for many patients.

A few studies have described the association between radon and different histological subtypes of lung cancer showing a higher radon-related risk for small cell lung cancer and squamous cell carcinoma compared to adenocarcinoma (Darby et al. 2005; Krewski et al. 2006; Taeger et al. 2006; Ramkissoon et al. 2018; Li et al. 2020; Rodríguez-Martínez et al. 2022). In terms of clinical characteristics of the patients and pathological data from the tumours related to radon, however, the available data is still scarce. Thus, to date the profile of lung cancer or the clinical characteristics of the subjects with radon-associated lung cancer is still not well defined.

With the development of molecular oncology, several oncogenic genomic alterations have been described in the group of non-small cell lung cancer (NSCLC) with a primary role in lung cancer pathogenesis (Thai et al. 2021). These alterations result in an aberrant activation or upregulation of highly relevant cancer pathways promoting cell growth and survival (Cancer Genome Atlas Research Network 2014). The main groups of molecular alterations are chromosomal rearrangements (e.g., *ALK, ROS1, RET,* and *NTRK1/3*) and specific genomic mutations (e.g., *EGFR, BRAF, MET,* and *HER2*).

The discovery of these alterations has led to a new arsenal of targeted therapies specifically directed against these constitutively activated cancer pathways. Indeed, nowadays, the lung cancer oncologist determines these molecular alterations in the tumour tissue in clinical practice and uses this information to guide the treatment selection. This approach of targeted therapies has transformed outcomes for several of these molecular subgroups (Planchard et al. 2018). Research in this field of lung cancer has increased exponentially and numerous molecular alterations have been identified and are currently studied as potential therapeutic targets (Thai et al. 2021).

While somatic mutations of *EGFR, BRAF, HER2, MET* and chromosomal rearrangements of *ALK, ROS1, RET* are responsible for lung cancer, risk factors for these genomic alterations have not yet been identified. In addition, most of these alterations have been described mainly in non-smoking population with NSCLC, in which radon is considered as one of the major risk factors (WHO, 2009). These molecular groups also have different clinical phenotypes, therapeutic implications and cancer prognosis compared to lung cancer related to smoking (Thai et al. 2021).

The advances made in molecular oncology, particularly with the comprehensive genomic characterization of lung cancer, resulted in a new multidisciplinary line of research to study whether radon exposure can have an impact on the genomic and clinical profile of the different subtypes of NSCLC. So far, three small preliminary studies (Taga et al. 2012; Ruano-Ravina et al. 2016; Mezquita et al. 2019) tested the potential association between residential radon and certain molecular subgroups of lung cancer in patients with NSCLC. It was observed that patients with tumours harbouring *ALK*-rearrangements, *EGFR* and *BRAF*-mutations had indoor radon levels above the WHO recommendation of 100 Bq/m^3^, particularly for *ALK*-rearrangement. The statistical power, however, was poor because of the small sample sizes.

Based on this preliminary evidence, new studies currently ongoing are further investigating the potential relationship between radon exposure and specific molecular subgroups of lung cancer. The Radon France is to date the largest ecologic study that has evaluated the indoor radon estimation and molecularly defined lung cancer subpopulation. The correlation between radon exposure areas in France based on the official French Radon map and the molecular alterations from the French National Cancer Institute was retrospectively tested in a cohort of 116,424 NSCLC cases (Mezquita et al. 2018). The prevalence of driver oncogenic alterations in patients with lung cancer, particularly *EGFR, BRAF, HER2* and *ROS1*, was significantly higher in high exposure areas.

Later, the role of indoor radon concentration estimated by birthplace was studied in case of 3994 patients with NSCLC from the Biomarkers France cohort, with access to clinical data. Based on geostatistical modelling from the Radon National Map (Ielsch et al. 2010), higher prevalence of driver oncogenic alterations was observed in patients born in high-radon areas, particularly in cases with *ALK* fusion and *EGFR* mutation. However, no significant differences were found after adjustment on age, gender, and smoking (Mezquita et al. 2021). Thus, the link between radon exposure and specific molecular alterations remains unclear.

Other types of studies exploring the molecular profile of lung cancer associated with radon exposure are the preclinical (in vitro and in vivo) and translational research in humans focused on radon and molecular epidemiology (Rosenberger et al. 2018; Gomolka et al. 2022). Indeed, the molecular mechanism of radon-induced carcinogenesis still remains unknown (Robertson et al. 2013). Alpha particles emitted by radon progeny induces a wide variety of cytotoxic and genotoxic effects, among which genetic mutations have been described, including point deletions or substitutions, and high chromosomal instability with chromosomal rearrangements, among others. However, these were generally studied in peripheral blood lymphocytes, and not in lung cancer tissue.

### Priorities for new research activities

Various studies are attempting to improve the scientific evidence on the relationship between radon exposure and molecular subgroups of lung cancer in the RadoNorm consortium. To build a radon-associated signature, the studies are assessing the link between the clinical, pathological, and molecular profile of NSCLC and the exposure to radon. For this purpose, an animal model and two different cohorts of patients are studied. Radon-induced lung cancer in rats is retrospectively characterized pathologically and molecularly, likewise lung cancers in Wismut miners who were exposed to different radon concentrations are investigated molecularly (Rosenberger et al. 2018; Gomolka et al. 2022). A prospective study is also ongoing, since April 2022, with the aim to characterize the clinical, pathological, and molecular profiling of patients with lung cancer exposed to indoor radon in Europe (The Bioradon—*EORTC 1920* LCC).

With the advances in sequencing technology, it is nowadays possible to identify thousands of somatic mutations, which makes it possible to identify molecular signatures with novel data-analytical methods. Using computational genomics, this research focusing on lung cancer induced by radon exposure in rats can provide a better understanding of the specific genomic alterations and the underlying molecular mechanisms. In addition, similar data obtained in humans can improve the knowledge of the radon-related carcinogenic processes and might result in the identification of a radon-associated signature.

Finally, an unresolved matter is the synergistic effect of radon exposure in combination with exposure to other relevant carcinogens such as tobacco smoke (NRC 1999b). It is well known that both are carcinogens and both induce oxidative stress and DNA damages but the mechanisms explaining the observed strong synergy are lacking. It is also unknown whether combined exposures leads to specific signatures and clinical consequences in patients (Tomasek 2011). More specific studies focussing on combined exposures, also with other environmental pollution (e.g., air pollution, asbestos), should be carried out to provide a scientific basis of the synergistic mechanisms and to improve our understanding of lung cancer development in general.

## Experimental biology

### Radiation quality dependent repair

Inhalation of radon and its progeny predominantly result in the exposure of cells in the lungs to alpha particles. The traversal of an alpha particle through a cell nucleus results in the deposition of energy along its track. This local energy deposition leads to relatively large quantities of clustered damage; complex DNA damage comprised of closely spaced lesions in the DNA molecule (Hada and Georgakilas 2008; Eccles et al. 2011; Hagiwara et al. 2017) that represent a major challenge for the cellular repair machinery. As especially the repair of induced DNA double strand breaks (DSBs) is crucial to prevent cell death, it is important to realize that alpha-particle-induced DSBs are much slower repaired (Nikitaki et al. 2016; Timm et al. 2018) than DSBs induced by sparsely ionizing radiation.

The observed delayed repair of ‘complex DSBs’ is probably related to the finding that a subset of these DSBs is refractory to repair by the nonhomologous end joining (NHEJ) pathway but depend on the homologous recombination (HR) pathway for repair (Frankenberg-Schwager et al. 2009; Zafar et al. 2010; Gerelchuluun et al. 2015; Roobol et al. 2020). These observations link repair of clustered damage to cell cycle progression since HR only operates during the S/G2 phase of the cell cycle. DSB repair capacity and accuracy determines for a large part the fate of a cell.

In low-dose exposure scenarios, the extensive adverse biological consequences of the traversal of a single alpha particle through the nucleus of a cell are highly relevant since, at the cellular level, such a single ‘hit’ represents the lowest possible exposure level. Exposure of Chinese hamster ovary cells to alpha particles with various energies revealed that a single alpha particle track can induce up to 0.39 lethal lesions. However, as the authors noted, the observed lethality might vary significantly with cell morphology as well as with cell type (Tracy et al. 2015). Especially upon chronic inhomogeneous exposures, local induction of cell death is likely to occur and will influence the biological consequences of exposure (Chen et al. 2020). On one hand, an inactivated cell can no longer contribute to cancer initiation or progression, while on the other hand the increased cell proliferation (of non-lethally exposed cells) required to replace the inactivated cells might elevate mutation frequency and contribute to cancer development (Hazelton 2008; Madas and Balásházy 2011).

### Indirect effects of exposure

The biological consequences of alpha particle exposure are not restricted to cells experiencing a nuclear traversal as also neighboring, non-targeted cells, can be affected. The targeted cells elicit molecular signals which are received by the surrounding non-targeted cells, a phenomenon often referred to as the radiation induced bystander effect (RIBE). RIBE signaling may have a large variety of biological effects that can be linked to multiple hallmarks of cancer (Heeran et al. 2019).

In contrast with the direct effects of IR, RIBE signaling seems not linearly related with dose and can already be observed at low levels of exposure. The magnitude of RIBE is influenced by radiation quality with a general trend of high LET radiation inducing stronger bystander responses per dose unit. These findings were recently confirmed in a co-culture system with primary human cells exposed to either alpha particles or X-rays, showing increased micronuclei, chromosomal aberrations, and genomic instability in unexposed bystander cells (Kanagaraj et al. 2019; Karthik et al. 2019). Strikingly, the RIBE signals of irradiated fibroblast did not affect the micronuclei formation in blood lymphocytes, suggestive of cell type specific signaling (Kanagaraj et al. 2019). However, human bronchial epithelial cells (BEAS-2B) displayed exacerbated detrimental effects after alpha particle exposure when co-cultured with non-irradiated human macrophage cells (Fu et al. 2016a, b), a detrimental form of the so-called radiation-induced rescue effect (RIRE, reviewed by Yu 2019). Beneficial RIRE effects whereby rescue signals from bystander cells protect irradiated cells have also been reported after α-irradiation (Chen et al. 2011; He et al. 2014; Lam et al. 2015a, b). Cyclic adenosine monophosphate (cAMP) membrane signaling, and the activation of the NF-kB pathway have been proposed as underlying mechanisms (Lam et al. 2015b).

Even one of the most commonly used procedures to assess cell survival after exposure, i.e. the colony forming assay, has been shown to be influenced by cellular communication (Adrian et al. 2018). The surviving fraction increased with the seeding density of irradiated cells, and under circumstances where irradiated cells could communicate with nonirradiated cells. Similarly, conditioned medium from densely seeded nonirradiated cells was shown to be associated with increased proliferation, DNA repair, and survival in A549 cells (Desai et al. 2014). The induction of ATF-2 transcriptional activity by autocrine soluble factors was identified as an important factor for the observed radioresistance.

Matsuya and co-workers (Matsuya et al. 2019) compared the cellular responses of chronic uniform exposure with heterogeneous exposures using insoluble particles containing radioactive cesium. Normal human lung fibroblasts and epithelial cells showed an increase of DNA damage in cells distal to the cesium particle, probably due to the induction of reactive oxygen species, whereas a reduced yield of DNA damage was observed in cells proximal to the source possibly due to rescue signals. These in vitro observations suggest a tradeoff between advantage and disadvantage signaling between cells under prolonged non-uniform exposure conditions.

The notion that the adverse effects of radiation on cells are modulated by the presence of non-exposed cells in their vicinity, and vice versa, implicates that the size and volume of exposed tissue affect the biological response to and therewith the potential consequences of exposure. Consequently, a change in exposure field will alter the ratio and distance between exposed and non-exposed cells and therewith the extent of bi-directional signal exchange. Early observations of radiation-induced field size effects (RIFSE) were reported after beta radiation of skin. The larger the field area, the higher the incidence of skin disorders, even with the same absorbed skin dose (Coggle et al. 1984; Peel et al. 1984; Hopewell et al. 1993). Recently, Ojima et al. (2021) was able to demonstrate RIFSE utilizing an X-ray microbeam setup to expose human cell populations with different exposure field sizes. Shortly after 1 Gy exposure, significantly more nuclear repair foci (53BP1) were observed in cells present in the larger exposure fields compared to cells in the smaller fields. This difference became even more pronounced during cell recovery and persisted for at least 48 h. In addition, the number of foci tended to decrease faster in cells in contact with non-irradiated cells at the border of the exposure field. In this area, the number of proliferating cells was also higher after 24 h and 48 h recovery when compared to the central region of the larger exposure fields, possibly due to migration of unexposed cells towards the exposed area.

Extensive cell migration was also reported after non-uniform radiation fields applied in microbeam radiotherapy in which the migration of stem cells likely explains the observed tissue-sparing effect (Slatkin et al. 1992; Crosbie et al. 2010; Fukunaga et al. 2019, 2020). Also repeated radon exposure of human bronchial epithelial cells (BEAS-2B) led to increased cell migration (Xu et al. 2019; Chen et al. 2020) and coincided with reduced apoptosis, loss of cell–cell adhesion, and signs of epithelial to mesenchymal transition.

### Priorities for new research activities

Cell lines show large differences in both intrinsic radiosensitivity as well as in relative biological effect (RBE) often accessed by comparing cell survival after high- and low LET radiation (Flint et al. 2021). The repair capacity of cells to deal with the induced damage is probably the main reason for the observed differences (Chistiakov et al. 2008; Borràs-Fresneda et al. 2016). However, a detailed understanding on the processes and pathways involved in the repair of the complex DNA damage induced by alpha particles is still lacking. Technological limitations are mainly responsible for the constrained activity in this field of radiation research. However, recently developed high-throughput alpha particle irradiation systems (Stanley et al. 2020) will allow genetic screens and are expected to provide a faster and deeper understanding of the repair pathways involved in the removal of complex DNA damage.

Besides these probably DNA repair capacity-driven differences in radiosensitivity of cells, it has become clear that also the cellular environment of exposed cells plays an important role in the ultimate consequence of exposure. Especially in case of inhomogeneous exposures, where irradiated cells are surrounded by unexposed (or less-exposed) cells, the impact of the tissue environment might be substantial. A central question is whether these ‘bystander’ factors have predominantly a detrimental or beneficial effect. The answer will probably depend on the type of exposed cells as well as on the type of cells in their neighborhood. Future research should, therefore, investigate the consequences of exposure for the relevant cell types and preferentially in their physiological tissue environment.

The bronchial epithelium is the principal target tissue for alpha-radiation due to inhaled radon progeny. Within this tissue the basal and secretory cells have the potential of cell proliferation (Leach and Morrisey 2018), and thus represent the most relevant cell types for cancer initiation. Although this is recognized in the field of dosimetry and risk modelling (ICRP 1994; ICRU, 2012), surprisingly little is known about the actual biological effects of alpha particle exposure on these two cell types. Within the bronchial epithelium, these cells are mostly non-proliferative and only start dividing upon tissue damage (Leach and Morrisey 2018). This is in sharp contrast with cell lines usually applied in radiation research, which typically display exponential growth due to permanent proliferative signals.

With the development of 3D cultures of primary human bronchial epithelial cells and organ-on-a-chip technology, in vitro model systems became available to study the effects of (inhomogeneous) alpha particle radiation in a physiologically relevant environment. In these models, basal and secretory cells are present together with ciliated cells to form a tissue with strong cell adhesion and intercellular communication closely resembling human bronchial epithelium (Boei et al. 2017; Hiemstra et al. 2018; Upadhyay and Palmberg 2018). Analysis of DNA repair processes in the relevant cells under in vivo-like conditions and unraveling the role of intercellular interactions on the fate of exposed cells will provide a more realistic insight in the consequences of localized exposure of lung tissue. Another source of valuable information regarding the effects of inhomogeneous alpha particle exposures are studies investigating the effects of targeted alpha therapy (Guerra Liberal et al. 2020; Li et al. 2022) in particular, when clinical studies are combined with more fundamental research.

## Dosimetry at small spatial scales

Internal microdosimetry of radon progeny in the lung comprises problems of internal dosimetry, such as the spatial and temporal distribution of alpha-emitting ^218^Po and ^214^Po activities on bronchial airway surfaces, the microdistribution of basal and secretory cells in bronchial epithelium, which are the most relevant cell types for bronchial carcinomas (ICRP, 1994; ICRU, 2012), and microdosimetry, such as the distribution of energy deposition in individual cell nuclei (Hofmann et al. 2020).

Factors contributing to radionuclide and target cell distributions are (1) inter- and intrasubject variability of radon progeny deposition (Hofmann et al. 2010) and mucociliary clearance (Hofmann and Sturm 2004) in an asymmetric stochastic airway structure (Koblinger and Hofmann 1985), (2) distribution of basal and secretory cell nuclei across the bronchial epithelium (Mercer et al. 1991) and diameter-related thickness of the epithelium (Hofmann and Winkler-Heil 2020), and (3) microdistribution of radon progeny surface activities, such as local accumulations at bronchial airway bifurcations due to enhanced deposition (Balásházy and Hofmann 2000) and reduced mucociliary clearance (Farkas 2020).

Microdosimetry refers to the distribution of energy deposition in micro-meter sized volumes, which is typically the nucleus of a cell. Factors affecting the variability of energy deposition of alpha particles in cell nuclei are (1) the hit probability, i.e. the probability of hitting a cell nucleus due to the limited range of alpha particles, and the related frequency of single and multiple hits (following Poisson distribution), and, (2) in the case of a hit, the variability of energy deposition of traversing alpha particles, caused by random track lengths of alpha particles in spherical cell nuclei (crossers) or incomplete traversal (stoppers), and the distribution of alpha particles ranges and the related LET dependence of intersecting tracks (Bragg curve). In the case of alpha particles, the specific energy distribution, *f*(*z*) can be approximated by the track length distribution for a given LET and the lineal energy distribution, *f*(*y*) by the LET distribution (Hofmann et al. 2000).

The microdosimetry of internal alpha-emitters (volume and surface sources) was developed by Roesch (1977), who extended the fundamental concept of external microdosimetry (Rossi 1968) to internally deposited alpha-emitting radionuclides. This analytic code has been applied to inhaled radon progeny deposited on cylindrical bronchial airway surfaces by Hui et al. (1990), Sedlák (1996) and Li and Zheng (1996). A different approach has been chosen by Aubineau-Laniece et al. (2002) and Fakir et al. (2005), who developed a Monte Carlo code for the calculation of specific energy spectra in bronchial airway bifurcations based on the analytic RADONA code of Caswell et al. (1994).

In the case of radon progeny alpha particles in bronchial airways, two microdosimetric parameters have been proposed to describe the fluctuation of energy deposition in cell nuclei: (1) the hit probability and the frequency of cellular hits following a Poisson distribution (Crawford-Brown and Shyr 1987; Caswell et al. 1994; Truta-Popa et al. 2011) and, (2) specific energy distributions for single hits or for multiple hits at a given absorbed dose (Hui et al. 1990; Sedlák 1996; Li and Zheng 1996; Fakir et al. 2005). The hit probability concept considers the variability of cellular hits, while energy deposition is expressed by the mean specific energy. The hit concept is especially relevant for low level radon exposures, which are characterized by a small number of cells affected, i.e., small hit probabilities.

**Fig. 1:**
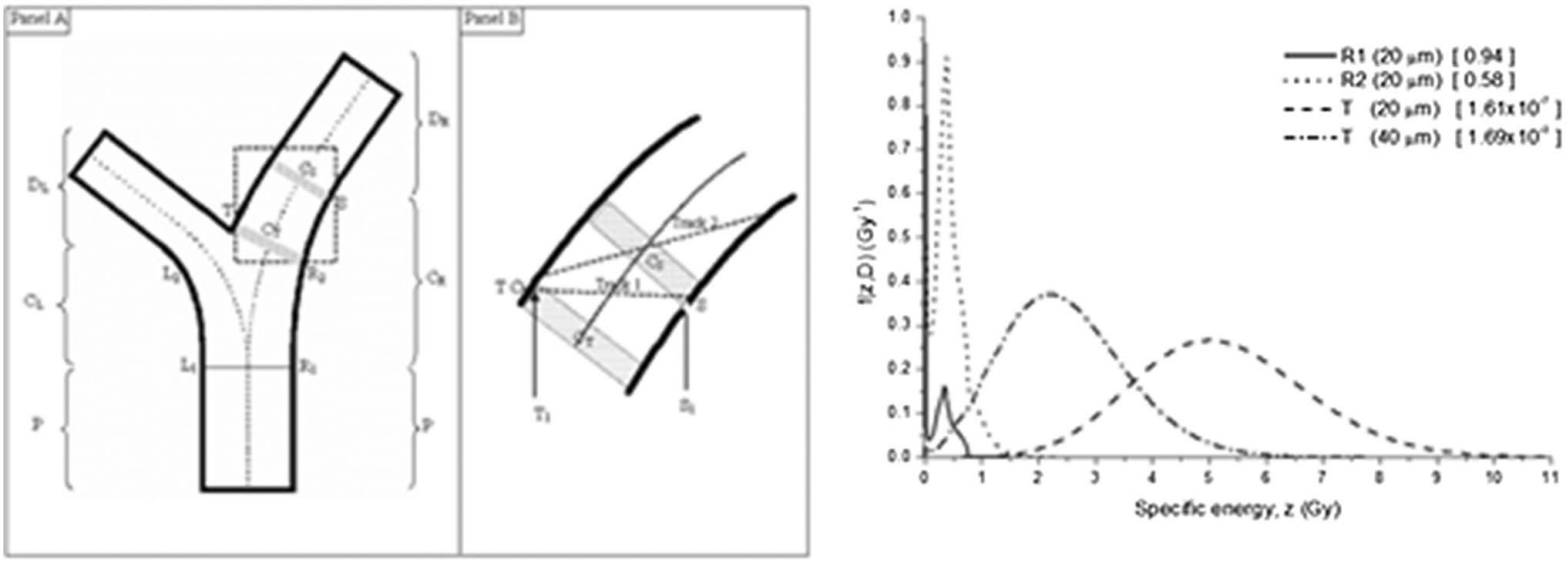
Dose-dependent specific energy spectra *f*(*z*;*D*) in three different target locations at bronchial airway bifurcations for non-uniform radon progeny surface activities, normalized to a cumulative exposure of 20 WLM (residential radon exposures). The numbers in parenthesis are the probabilities of zero events, indicating the fraction of cells not hit at all

For comparison, the specific energy distribution concept includes both the variability of cellular hits and the variability of energy deposition in cell nuclei. At low radon exposures, specific energy distributions in basal and secretory cell nuclei result from single energy deposition events. However, in the case of radon progeny accumulations at bronchial airway bifurcations, cells located directly at the carinal ridge will receive multiple hits even at low exposure levels (Fakir et al. 2005). This is illustrated in Fig. 1 for three selected cellular sites, along the cylindrical airway (R1), at the transition zone of the bifurcation (R2), and directly at the carinal ridge (T). Although the specific energy distributions in R1 and R2 differ in the number of alpha particle hits, they are still produced by single hits. For comparison, the two specific energy distributions in T for two different depths in tissue differ also in the number of hits received, but here the differences in multiple hits lead to distinctly different specific energy distributions.

Several microdosimetric approaches have been proposed to establish a relationship between hit probabilities or specific energy distributions and cellular radiobiological effects, such as hit-related models or effect-specific track length models. The basic hypothesis of hit-related models is that biological effects are related to the fraction of cells hit and the related Poisson distribution (Truta-Popa et al. 2011). In effect-specific track length models, the random intersection of cell nuclei and the multiplicity of cellular traversal are related to effect-specific probabilities per unit track length (PPUTL) as functions of LET for specific radiobiological effects, such as cell killing or oncogenic transformation (Hofmann et al. 2000).

**Fig. 2:**
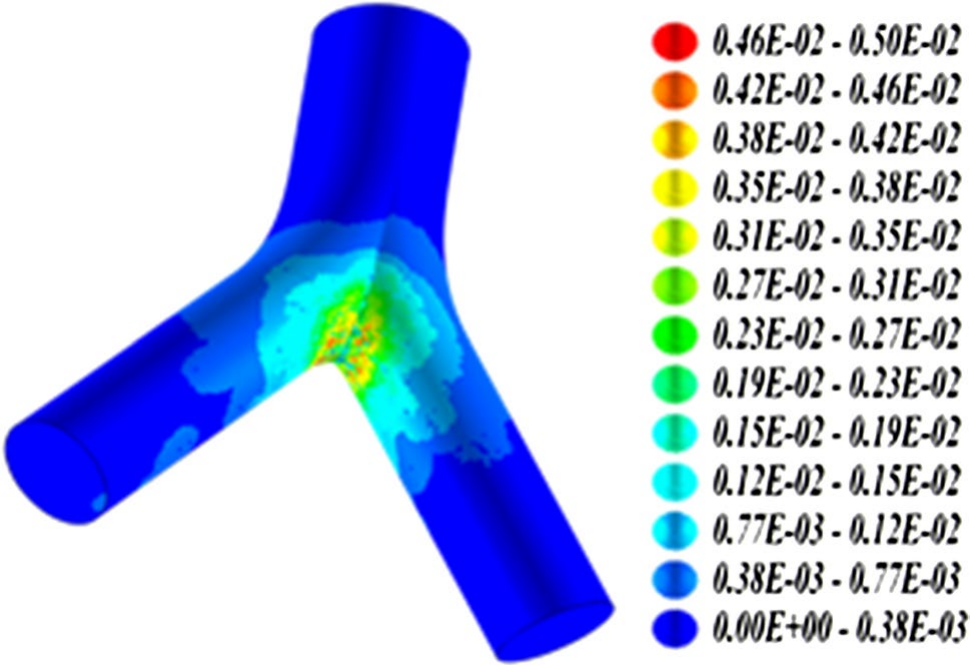
Distribution of transformed basal and secretory cells in a bronchial airway bifurcation model irradiated by radon progeny alpha particles based on direct hits plus indirect contributions of bystander cells

The application of the effect-specific track length model of Hofmann et al. (2000) to radon progeny accumulations at bronchial airway bifurcations (see Fig. 1) is illustrated in Fig. 2 (Szőke et al. 2012), demonstrating that the carcinogenic risk is significantly higher at bronchial bifurcation sites. Although the number of alpha particle emissions at the carinal ridge is about two orders of magnitude higher than along the cylindrical parts, the number of transformed cells is only about one order of magnitude higher due to the compensating effect of increased cell killing.

At low doses of alpha particles, where cells are traversed only by single alpha particles, specific energy distributions and the number of cells hit are the same for both uniform and non-uniform dose distributions. At bronchial airway bifurcations, cells located at the carinal ridge experience already multiple hits even at low average doses, thereby reducing carcinogenic risk by cell killing. On the other hand, these cells are concentrated around the hot spot may form a cluster of potentially malignant cells, thereby acting as a stimulus for further carcinogenic growth via cellular interactions (Fakir et al. 2009). Thus, with respect to the partial organ exposure aspect, cellular doses at airway bifurcations should be weighted by the density distribution of cells hit as a physical measure of inhomogeneity as well as by the survival probability for single and multiple hits as a biological measure of inhomogeneity.

### Priorities for new research activities

Microdosimetry refers to energy deposition in individual cell nuclei at a given point in time and the production of cellular biological effects, such as oncogenic transformation or cell killing. However, the initial physical response may be modified by simultaneously or subsequently acting biological mechanisms, such as non-targeted effects (Fakir et al. 2009) or adaptive response, genomic instability and induced cellular division (Truta-Popa et al. 2008). Consequently, microdosimetric energy distributions should be supplemented by cellular and tissue-specific biological mechanisms to predict lung cancer risk. Thus, future research should quantify such relevant biological factors and define their interaction with the initial energy deposition events, e.g., in terms of biological correction factors.

At typical indoor radon exposures, bronchial target cells hit by alpha particles are relatively uniformly distributed and widely spaced across the bronchial epithelium, except for the large bronchial airway bifurcations, where cells hit are more densely clustered around the carinal ridge (Fakir et al. 2005). Likewise, the distributions of resulting radiobiological effects, e.g., cells transformed, killed, or hit but surviving, still reflect the spatial distributions of the initial energy deposition events (see Fig. 2). Thus, future research should quantify the distributions of interaction distances between energy deposition events or resulting specific biological effects by clustering methods, such as dendrograms, nearest neighbour distributions or proximity functions to determine a spatial heterogeneity index.

In the case of low indoor radon exposures, where individual cellular energy deposition events are widely spaced in time, the temporal distribution of energy deposition events, and hence dose rate, will affect the resulting biological response, which may be accounted for by the dose and dose rate effectiveness factor (DDREF, for review see Lowe et al. 2022). If an already hit cell in bronchial epithelium is hit again by an alpha particle after about 30 days, which is the average lifetime of lungs cells (Adamson 1985), then the initial DNA damage in that cell may have already been repaired or that cell may have been replaced by a new cell by division or apoptosis. This suggests that the crucial radiation quantity for cellular effects in a protracted exposure, is the dose per cell cycle (Fakir et al. 2006). Thus, future research should quantify the temporal distributions by clustering methods of energy deposition events or resulting specific biological effects to determine a temporal heterogeneity index.

## Models linking effects at different levels of biological organization

Modelling the effects of ionizing radiation aims to identify quantitative links between measurable quantities upon radiation exposure and to provide insights into the mechanisms of action. The system of radiation protection focuses on the effects at the population level, while a large part of data is obtained from experiments at the cellular level. Therefore, there is strong need for mathematical models that links the effects observed at different levels of biological organization. If they are appropriately validated, these models can be used to estimate risks for low doses based on epidemiological data at high doses, and for exposures different from available epidemiological studies. The most recent UNSCEAR Report devotes an entire chapter on modelling of cancer mechanisms in its Annex C (2021), and a significant part deals with radon studies. Here, we focus on two types of models and as an example on how they explain inverse exposure-rate effects.

The most frequently applied model in radon carcinogenesis studies is the Two Stage Clonal Expansion (TSCE) model which provides a mechanistic framework for lung cancer mortality and exposure (Rühm et al. 2017). An advantage of this model is that its parameters can be derived from fits to epidemiological cohorts and from biological input, while the drawback is that it does not consider the spatial dose distribution within the lungs. The other types of models to be discussed here are aiming to make only one step by estimating the effects of radon exposure at the tissue level based on data at the cellular level. The advantage of these models is that they can consider the highly heterogeneous spatial dose distribution, while a major drawback is that until very recently their predictions were difficult to be compared with experimental data.

The TSCE model and its application to epidemiological data of lung cancer upon radon exposure has been recently reviewed (Rühm et al. 2017; UNSCEAR, 2021). In this model, normal cells can be initiated resulting in a proliferative advantage compared to their normal counterparts. Initiated cells can clonally expand, and they become the target of a second event called transformation. Finally, the transformed cells develop into a clinically detectable cancer and may cause death of the individual. In case of radon exposure, exposure rate is the independent variable, and not dose rate, and therefore, the spatially heterogeneous dose distribution in the bronchial airways is not considered.

In general, exposure to ionizing radiation can affect both the transitional steps (initiation and transformation) and clonal expansion (promotion). Most studies, however, show that radon exposure is primarily a promoting agent with lower or no effect on initiation and transformation (see e.g., Table 2 in Rühm et al. 2017; or Table 9 in UNSCEAR, 2021). These studies also show that the slope of the clonal expansion rate exposure rate function decreases with exposure rate and even levelling off (i.e., the slope equals zero) in many cases at high exposure rates. This nonlinear response of clonal expansion rate to exposure rate implies an inverse exposure rate effect.

Bystander effect is one of the biological mechanisms proposed to explain the nonlinear relationship between clonal expansion rate and exposure rate, and so inverse-exposure rate effect. Eidemüller et al. (2012) showed that bystander effect provides a plausible explanation for the observation, because the exposure response is amplified in the neighbourhood of the small fraction of hit cells via bystander signals at low exposure rates, but this effect saturates by increasing exposure rate because the fraction of hit cells also increases and approaches 1. They also estimated the fraction of hit progenitor cells at the exposure rate where the slope of the clonal expansion rate curve changes and found that it is compatible with the fraction of hit cells in in vitro experiments, where the contribution of bystander effects diminishes. In this estimation, however, the heterogenous activity distribution of radon progeny was not considered. Bystander effect as an explanation for inverse exposure-rate effect was also proposed earlier by Brenner and Sachs (2002, 2003), their bystander model, however, does not necessarily provide a better fit than a linear relative risk model with adjustment for age at exposure and attained age (Little 2004).

Considering the heterogeneous dose distribution in the bronchial airways, one of the most important questions is what the effects of locally high doses in the deposition hot spots are. One line of research focuses on the consequences of chronically high cell death rate in the deposition hot spots working with the hypothesis that maintaining tissue functions requires an equilibrium between cell killing and cell reproduction. This hypothesis is not applicable for acute or short-term exposures. Here, two major endpoints are considered (1) mutation induction and (2) induction of progenitor cell hyperplasia.

In one of these studies (Madas and Balásházy 2011), the contributions of two mechanisms to mutation induction was compared. Besides mutations induced at the site of DNA damages, the rate of spontaneous mutations also increases with the increase of cell division rate (Hazelton 2008). Alpha-particles can increase mutation induction rate in both ways. It was found that in the deposition hot spots most of the mutations occur due to the increased cell division rate, and not due DNA damages directly leading to mutations. However, there is a synergistic effect between these two mechanisms. An important implication is that mutations induced by radon exposure are not necessarily different from those of spontaneous origin.

The study shows that the same effect of ionizing radiation can be seen differently at different levels of biological organization. At the cellular level, dead cells cannot contribute to mutagenesis (see also Fig. 2 and related discussion), while cell killing increases the mutation rate in the neighbouring cells if simple control mechanisms at the tissue level are considered. In addition, the reduction of cell division rate also reduces the mutation rate, and as such radiation responses resulting in lower cell division rate can be considered as adaptive responses to chronic exposure to radon progeny. One of the mechanisms reducing the cell division rate is the increase of progenitor cell number, as less divisions are required if there is a larger pool of cells capable of division. Based on histological (Auerbach et al. 1961) and experimental evidence (McDowell et al. 1979) as well as considerations in mathematical biology (Lander et al. 2009), it was proposed that chronically high exposure to radon progeny can induce basal and goblet cell hyperplasia in the deposition hot spots (Madas and Balásházy 2011; Madas 2016b; Madas and Drozsdik 2018).

The induction of progenitor cell hyperplasia provides an alternative explanation for inverse exposure-rate effects. Supposing that the measure of hyperplasia, i.e., the additional number of progenitor cells monotonically increases by exposure rate, one can see that the same cumulative exposure results in a higher cumulative tissue dose if there is a longer exposure with lower exposure rate than if there is a shorter exposure with higher exposure rate. Considering that hyperplasia may also reduce the mutation induction rate upon the same tissue dose, the adaptive effect of hyperplasia is even stronger.

### Priorities for new research activities

The enormous challenge to reach the ultimate goal of modelling originates from the width of levels of biological organization to be covered: from experiments at the cellular level to epidemiology at the population level. Development and validation of biophysical models at the tissue level, which may be the most relevant level regarding carcinogenesis (Soto and Sonnenschein 2011), is a crucial step towards the goal of linking experimental and observational data. It can also help the integration of TSCE-based mechanistic models with models considering dose heterogeneity, which was not really successful previously due to the high number of unknown parameters (Madas and Varga 2014; Drozsdik and Madas 2019).

Another important task is to set up models which can predict the molecular characteristics of radon-induced lung cancer including the mutation spectrum. While alpha-particle exposure results in a typical genomic alteration spectrum with large deletions (Robertson et al. 2013), they may not be seen in radon-induced lung cancer cells if the major route of mutation induction is the increased cell division rate. Further development of existing models (e.g., Madas and Balásházy 2011) can be used to estimate the fraction of mutations characteristic of those directly induced by alpha-particles and those of spontaneous origin. Integration of these models with models of the cellular effects of ionizing radiation can result in further improvement.

It is an important question whether biophysical models which consider dose heterogeneity can predict the sites of neoplastic or preneoplastic lesions in humans and animals. Here, both animal and human studies provide opportunities for comparison of model predictions with clinical observations, potentially linking the dose distribution at the tissue level to the spatial distribution of molecular and clinical characteristics of radon-induced lung cancer. In the long term, it would be interesting to have models which are able to predict the probability of different histological subtypes of radon-induced lung cancer.

Finally, an important line of modelling aims to quantify those changes which modulate the biological system before it is exposed to radon progeny. This includes the modelling of the effects of smoking and different lung diseases on and the age dependence of the anatomy, physiology, and tissue architecture of the bronchial airways. These may affect not only the biological and health effects of radon exposure, but also the relationship between exposure and absorbed doses.

## Discussion and outlook

Considering the contribution of radon exposure to the natural background radiation and its association with lung cancer, it is highly important to better quantify the related risk. To achieve this goal, it should be further clarified whether radon can cause other diseases than lung cancer, and what are the health risks by radon in children or persons at young ages. An important step can also be the shift of focus in radon epidemiology from exposure–response to dose–response relationships. This requires the development of dosimetric protocols providing data on absorbed organ doses in the lungs and in other organs. These protocols may make use of recent in vivo measurements of activity distributions upon radon exposure (Papenfuß et al. 2022). Dosimetry may also allow to quantify the effects of age (including children) on absorbed doses upon a given radon exposure providing more detailed information on doses in epidemiological cohorts.

To reduce uncertainties, the better understanding of the combined effects of radon and smoking is also important. Radon is related to up to 14% of lung cancer cases (WHO 2021) as leading cause in lifelong non-smokers, and synergistically increases the risk in current smokers and ex-smokers. Identification and differentiation of lung cancer profiles related to radon exposure and smoking could be a crucial step leading to a more precise risk assessment. Modelling how smoking affects absorbed doses and dose distributions can also help to improve dosimetric protocols, and thus reduce uncertainties in epidemiological studies.

To extend the risk assessment towards lower exposures, the understanding of the mechanisms involved in radon carcinogenesis is required. Integrating epidemiological, clinical, pathological, and molecular oncology data to obtain a radon-associated signature may help to identify radon-related cancer pathways and establish hypotheses on lung cancer pathogenesis in non-smokers and synergistically in smokers. In the future, the comprehensive characterization of lung cancer related to the exposure to radon might also improve the patients’ care and promote cancer prevention strategies. Furthermore, understanding the molecular mechanisms provides valuable input for mathematical models of radon carcinogenesis, which then can provide risk estimates for low exposures.

The advances made in the development and application of 3D cultures of primary human bronchial epithelial cells and organ-on-a-chip technology can help to identify new and corroborate existing biomarkers, and to understand their role in early stages of carcinogenesis. With particular attention to alpha-particles and tobacco exposure, this technology may help to identify risk factors for somatic mutations and chromosomal rearrangements most frequently observed in NSCLC. Besides experimental data, improved models of mutation induction can also help to identify what kind of mutations we can expect. In the long term, dose–response relationships for some mutations and chromosomal rearrangements can also be established if biophysical models are appropriately validated in air-liquid interface models.

3D cultures of primary human bronchial epithelial cells can also provide useful input to TSCE-based mechanistic models and validation data for biophysical models. These experimental systems are the simplest that can mimic the tissues, where the effects of heterogeneous dose distribution can be studied. Therefore, modelling efforts have to focus on the effects of locally high doses and gradients in doses, and on predictions which can be compared with quantities measured in these experimental systems. As observing clonal expansion and hyperplasia may require longer follow-ups than possible, modelling should aim to identify endpoints characterizing the early changes in tissue architecture including cell division rate and cellular composition.

Identifying biomarkers of radon-related pathways can help to track carcinogenesis along time with particular attention on the location of preneoplastic and neoplastic changes. It is an important question whether the highly heterogeneous dose distribution is reflected in the spatial distribution of any molecular changes. If these changes can be attributed to initiation or transformation, then TSCE-based models can also be improved. If data are available on the location of histological samples from humans or animal models, studies focusing on the tissue composition inside and outside the deposition hot spots can shed light on the potential role of progenitor cell hyperplasia in radon carcinogenesis. Here, development of mathematical models can provide estimates on the measure of hyperplasia as the function of local dose rate and exposure rate. The increase in computational power can also be used for more precise dosimetry to obtain better quantification of dose distribution in humans and in animal models.

Besides its importance related to radon exposure, understanding the mechanisms involved may have more general implications for radiation protection regarding the effects of heterogeneous dose distributions within an organ. Mathematical modelling may help to quantitatively translate the knowledge obtained from radon exposure to other exposures. These models will require input from experiments comparing the effects of homogeneous and heterogeneous dose distributions. These models can also improve risk assessment even if huge changes are not expected due to dose heterogeneity at this spatial scale.

An important question is how differences between the effects of homogeneous and inhomogeneous exposures can be considered in the system of radiation protection. It is important to note that radiation protection applies weighting factors to consider the effects of spatial and temporal variation in dose delivery. To some extent, tissue weighting factors allow to take into account the heterogeneity in dose distribution within the organisms. Radiation weighting factors are strongly related to the heterogeneity in energy deposition at the microscopic (subcellular) level. Even the effects of temporal variation in dose delivery are considered by a factor, the dose and dose-rate effectiveness factors. In line with this, a weighting factor considering the effects of spatial variation in dose delivery within the organs is relevant and one should aim to determine such a factor.

It is current practice in radiation protection that the dose delivered by alpha particles to a given tissue is assigned to all target cells. However, at low radon exposures, only a small number of cells in a given tissue volume are hit by alpha particles, which receive a relatively high energy, while most cells are not hit at all. Thus, low doses of alpha particles are characterized by a low number of cells actually hit and not by low cellular doses in all cells in that tissue volume, as would be the case after low LET exposures. Thus, with respect to the partial organ exposure aspect in the case of high LET radiations, the dose to a specific tissue should be weighted by the fraction of cells actually hit or by the hit probability for that tissue, to allow a realistic prediction of the health consequences.

Inhalation of radon progeny produces an extremely inhomogeneous dose distribution in the lungs, with only a few a percent in the alveolar-interstitial region (AI) region (about 960 g) compared to the bronchial (BB) and bronchiolar (bb) regions (about 40 g). Despite similar doses in the BB and bb regions, a clear majority of bronchial carcinomas has been observed in the BB region. While this inhomogeneity of dose and cancer distributions is currently not considered by ICRP (1994), where equal apportionment factors are assigned to each lung region, Winkler-Heil et al. (2015) proposed apportionment factors of 0.6:0.3:0.1, based on dose distributions for different exposure conditions and related lung cancer incidences. Thus, with respect to the partial organ exposure aspect, organs (e.g., lungs) should be subdivided into distinctly different dose regions, and these regions should be weighted by related biological factors such as the relative volumetric density of target cells or the pathologically observed lung cancer distribution.

Given the complexity of issues in radon epidemiology and radon dosimetry, a multidisciplinary approach is required to improve risk assessment and mechanistic understanding of carcinogenesis related to radon exposure. These will also help to gain knowledge on the effects of spatial variation in dose delivery in general, not just related to radon exposure. As exposure to ionizing radiation is a major epidemiological concern world-wide, the mechanisms how radon radiation act and the potential long-term health consequences need to be thoroughly investigated. These studies will also raise the awareness of this preventable but silent risk factor, which can be instrumental to promote radon national plans and strategies on cancer prevention.

## Footnotes

1 Working level month is the historical unit of exposure to radon progeny applied to the uranium mining environment. One WLM (equivalent to 3.54 × 10^–3^ Jh m^−3^) is defined as the cumulative exposure from breathing an atmosphere at a concentration of one working level (WL) for a working month of 170 h. A concentration of 1 WL is any combination of short-lived radon progeny in one litre of air that will result in the emission of 1.3 × 10^5^ MeV of alpha-energy (ICRP 2010).
